# The Gut’s Secret Code: Bowel Microbiota as a Biomarker for Adaptation

**DOI:** 10.3390/nu17132117

**Published:** 2025-06-26

**Authors:** Joanna Braszczyńska-Sochacka, Jakub Sochacki, Michał Mik

**Affiliations:** Department of General and Colorectal Surgery, University Clinical Hospital No. 2 of the Medical University of Lodz, 90-549 Lodz, Poland; k.sochacki.86@gmail.com (J.S.); michal.mik@umed.lodz.pl (M.M.)

**Keywords:** bowel microbiota, bowel adaptation, biomarker

## Abstract

**Background:** Chronic intestinal failure (CIF), most commonly caused by short bowel syndrome (SBS), necessitates complex care. This review explores the gut microbiota’s role in intestinal adaptation in SBS, examining its potential as both a biomarker and therapeutic target. SBS results from extensive small bowel resection, leading to malabsorption and dependence on parenteral nutrition (PN). Post-resection, the gut microbiota undergoes significant alterations. While the small bowel microbiome typically comprises Streptococcus, Veillonella, and others, SBS patients often exhibit increased Gram-negative Proteobacteria. Dysbiosis is linked to adverse outcomes like liver disease and impaired growth, but beneficial effects such as energy salvage also occur. Intestinal adaptation, a process of increasing absorptive surface area in the remaining bowel, involves acute, remodeling, and maintenance phases. Preservation of ileum and stimulation with the oral diet are crucial. Biomarkers are needed to predict success, with gut microbiota composition emerging as a promising non-invasive option. The precise mechanisms driving adaptation remain incompletely understood. **Conclusions:** GLP-1 and GLP-2 analogues show promise in enhancing adaptation and reducing PN dependence. Surgical rehabilitation aims to maximize intestinal absorptive capacity, while transplantation remains a last resort due to high complication risks. Further research is needed to fully elucidate the microbiota’s role and harness its potential in managing SBS.

## 1. Introduction

The adult human gut hosts a complex and highly diverse microbial ecosystem—collectively referred to as the gut microbiota—comprising over 1000 species from all domains of life, including Archaea, Bacteria, and Eukarya [1]. Functioning almost as a vital organ, this microbial community engages in multidirectional communication with other body systems through neural, endocrine, immune, and metabolic pathways [1]. The gut microbiota plays a pivotal role in host metabolism, immune regulation, maintenance of intestinal homeostasis, and defense against pathogenic organisms [1,2]. Its composition and functional activity are influenced by a range of factors, including diet, host genetics, environmental exposures, and medication use. Despite these influences, the gut microbiota typically demonstrates remarkable stability and resilience in healthy individuals [3].

Beneficial and Harmful Effects of Gut Microbiota

Beneficial gut microbes play a crucial role in the digestion of complex carbohydrates and dietary fibers, producing short-chain fatty acids (SCFAs) such as butyrate, propionate, and acetate. These SCFAs serve as vital energy sources for colonocytes and modulate host metabolism and immune function [4]. Additionally, gut microbes synthesize essential vitamins (e.g., vitamin K and B vitamins), metabolize xenobiotics and pharmaceuticals, and maintain the structural integrity of the intestinal mucosal barrier [5].

In contrast, disturbances in this balanced microbial community—referred to as dysbiosis—can result in the overgrowth of pathogenic bacteria, production of harmful metabolites, and impaired barrier function. Dysbiosis has been associated with a broad spectrum of conditions, including inflammatory bowel disease, metabolic syndrome, liver disorders, infections, and neurological diseases [2]. Common contributors to dysbiosis include antibiotic use, dietary alterations, infections, and environmental exposures, all of which can adversely affect microbial diversity and functionality [2].

Microbiota Dysbiosis and Its Role in Bowel Syndromes

In bowel disorders such as short bowel syndrome (SBS), massive small bowel resection leads to changes in the intestinal luminal environment, including an acidic pH, increased oxygen levels, and disrupted enterohepatic circulation. These alterations modify the ecological conditions within the gut, shifting selective pressures that favor the growth of certain microbial populations while inhibiting others, thereby disrupting the overall microbial balance [2,3,6]. As a result, there is often an overrepresentation of facultative anaerobes—particularly Proteobacteria—alongside a marked decline in beneficial obligate anaerobes, such as members of the Clostridium clusters and the Bacteroidetes phylum. This dysbiosis can hinder intestinal adaptation, compromise nutrient absorption, and increase the risk of complications, including small intestinal bacterial overgrowth and D-lactic acidosis. Under normal conditions, the small intestine presents a challenging environment for microbial colonization due to the rapid movement of luminal contents, continuous exposure to digestive enzymes and bile, and the intermittent availability of nutrient substrates [1]. However, the physiological changes following resection can favor microbial shifts with important clinical implications. A deeper understanding of how these microbial communities evolve and interact with the host during intestinal adaptation is essential for the development of diagnostic biomarkers and targeted therapeutic strategies.

Objectives and Scope of the Current Review

This review aims to present a comprehensive overview of the role of the gut microbiota in intestinal adaptation following short bowel syndrome (SBS). It will examine the potential of the gut microbiota as a non-invasive biomarker for monitoring the progression of adaptation and the emergence of complications. Additionally, the review will explore emerging microbiota-targeted therapeutic strategies aimed at enhancing adaptation and clinical outcomes. By synthesizing current evidence on microbial composition, functional dynamics, and host–microbiota interactions, this review seeks to identify opportunities for personalized, microbiome-based interventions to support patient care and improve long-term outcomes in SBS. This is a narrative review based on a non-systematic literature search. A systematic review was not pursued due to the limited number of high-quality, comparable studies currently available in this emerging field. Relevant articles were identified through searches in the PubMed database using the keywords short bowel syndrome, intestinal microbiota, and bowel adaptation. The search was limited to English-language publications from the last 20 years (2004–2024), although key earlier studies were included if considered foundational. Studies were selected based on relevance to the topic, including both human and relevant preclinical (animal model) data. Additional articles were identified by screening the reference lists of included publications.

## 2. Intestinal Failure

Chronic intestinal failure (CIF) is a heterogeneous disease that impacts pediatric and adult populations worldwide and necessitates complex multidisciplinary care [7]. The most common cause of CIF is short bowel syndrome (SBS), a rare condition characterized by a remaining small intestinal length of less than 200 cm, typically resulting from surgical resections due to various diseases [8]. The prevalence of SBS is 1.4 cases per million people [9]. Based on the site of resection, three main anatomical types of SBS can be distinguished. Type 1 (end-jejunostomy or end-ileostomy) occurs when both the colon and the ileum (partially or completely) are resected. Type 2 (jejuno-colonic anastomosis) results from the removal of all or most of the ileum along with part of the colon. Type 3 (jejuno-ileal anastomosis) occurs when a segment of the terminal ileum remains in continuity with an intact colon while preserving the ileocecal valve [8]. Patients with SBS suffer from insufficient absorption of essential macronutrients, water, and electrolytes, necessitating intravenous supplementation to maintain health and nutritional balance [8]. Parenteral nutrition (PN) in SBS is a life-saving procedure for many patients. The goal of PN is the intravenous administration of nutrients. The parenteral mixture is a complex combination of amino acids, carbohydrates, fat emulsion, vitamins, and micro- and macroelements [10]. In the early postoperative period, the full nutritional requirement must be supplied parenterally until gastrointestinal function is restored and oral intake of fluids and solid foods becomes possible [10,11]. The extent of resection determines whether patients can potentially discontinue parenteral nutrition during the intestinal adaptation phase, which can last 2–3 years, while others may require full or partial parenteral nutrition for many years to maintain adequate nutrition and hydration [10,11].

### 2.1. Microbiota Alterations After Intestinal Resection

Various factors can impact the gut microbiome in patients with intestinal failure (IF), including gastrointestinal conditions, dietary patterns, and medications [8]. The specific composition of the SBS microbiota is closely related to post-surgery outcomes and adaptation [5]. The small bowel microbiome is typically dominated by species such as Streptococcus, Veillonella, Prevotella, Rothia, Haemophilus, Actinobacter, Escherichia, and Fusobacterium [1,12,13].

When analyzing compositional changes, most studies on patients with SBS have reported a notable increase in Gram-negative Proteobacteria, particularly Gammaproteobacteria and the Enterobacteriaceae family [6,12,14,15,16,17,18]. The fecal microbiota of SBS patients is modified, showing a high prevalence of Lactobacillus, a sub-dominance of Bacteroidetes, a significant depletion of Clostridium coccoides, and the absence of Clostridium leptum [19].

Some studies have included participants with ileostomies, allowing for longitudinal effluent sampling directly from the small intestine. While this offers methodological advantages, it is crucial to note that ostomy samples are exposed to the skin and the external environment, which may influence the microbial community [1].

In patients with CIF, gut microbial dysbiosis has been linked to negative outcomes such as liver disease, D-lactic acidosis, prolonged intestinal adaptation, decreased weaning capacity, and poor growth in children [8,9]. However, gut microorganisms have also been shown to have beneficial effects, including energy salvage and supporting adaptive mechanisms after gut resection in SBS patients [8,9,12].

### 2.2. Intestinal Adaptation and Rehabilitation: A Complex Process

**Figure 1:**
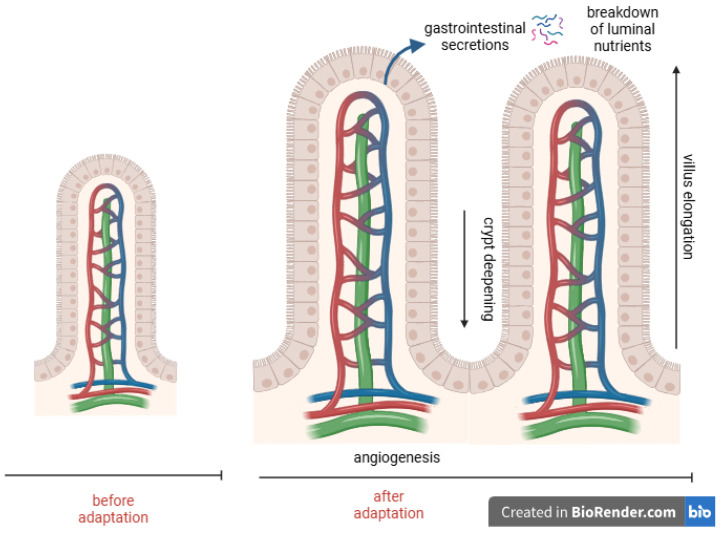
Functional and structural adaptation of the intestine after extensive resection.

The bowel adaptation is a physiological process in which the remaining small bowel increases its available surface area to compensate for the surgical loss of capacity through intraluminal stimuli from nutrients and gastrointestinal secretions (Figure 1) [20,21].

#### 2.2.1. Phases of Intestinal Adaptation

Clinically, adaptation becomes evident through the progressive tolerance of oral nutrition, which was previously intolerable at earlier stages. A fully developed adaptation response enables complete nutritional absorption from the gut, eliminating the need for supplemental parenteral feeding. Within hours of intestinal resection, increased expression of several immediate-early genes has been observed in the remaining bowel [22,23,24,25].

This process consist of three phases:

Phase 1 (Acute): Lasting up to four weeks after major surgery, characterized by loss of gastrin regulation and peak gastrointestinal losses. The primary complications during this phase include dehydration, electrolyte imbalances, and renal failure [26,27,28,29,30].

Phase 2 (Adaptation): May extend up to two years and includes anatomical and histological remodeling. During this stage, the lumen enhances absorption through villi elongation and crypt deepening. During this stage, the intestinal lumen enhances its absorptive capacity through villi elongation and crypt deepening. Additionally, a key adaptation of the small intestine is the slowing of intestinal transit, which prolongs nutrient–mucosal contact and improves absorption [25,30,31].

Phase 3 (maintenance): Involves proper oral nutrition, regular assessment of nutritional status, periodic monitoring of serum electrolyte levels, and, if necessary, correction of their concentrations through intravenous infusions [25,30,31].

#### 2.2.2. Role of Intestinal Anatomy

The preservation of anatomy plays a crucial role in the adaptation process. The increased ability of the colon to process nutrients allows for additional caloric absorption, serving as an extra mechanism of functional intestinal adaptation after massive resection. Ziegler and his team conducted a study on patients with short bowel syndrome, during which a fivefold increase in PepT1 (peptide transporter 1), responsible for di- and tripeptide transport in the colon, was observed [30,31]. The preservation of anatomy plays a crucial role in the adaptation process. The ileum demonstrates a higher adaptive potential than the jejunum. The composition and osmolarity of the digestive contents from the upper gastrointestinal tract differ in the jejunum and ileum, which directly impacts the mucosal adaptation of these sections. Resection of the distal small intestine is linked to a higher risk of diarrhea, particularly of a fatty nature, in contrast to resections of the proximal segment. Proximal small intestine resection offers a better chance of regaining gastrointestinal autonomy, as the structural and functional adaptive capacity of the ileum is greater than that of the jejunum, which only adapts functionally [32,33].

#### 2.2.3. Importance of Enteral Nutrition

Stimulation of the preserved segment of the small intestine with an oral diet is essential for maintaining the proper structure of the intestinal mucosa. The absence of nutrients in the intestinal lumen leads to mucosal atrophy and reduces the activity of digestive enzymes and nutrient transport, despite the provision of adequate energy, proteins, fats, and carbohydrates intravenously. Exclusive parenteral nutrition predisposes to villous atrophy, while the introduction of enteral/oral nutrition restores the microstructure and physiological function of the intestinal mucosa. Oral nutrition should be introduced as soon as possible after surgery [32].

#### 2.2.4. Individual Variability and Hormonal and Nutritional Mediator of Adaptation

Previous studies on adult SBS patients have examined individuals with highly diverse anatomical conditions, including variations in colonic continuity, presence or absence of the ileocecal valve, and ostomy status [6,8,13,19,34,35,36,37]. Not all patients respond in the same way throughout this process. To aid in determining and predicting success rates, biomarkers can be incorporated into patient management protocols [25].

As shown in Table 1 and Table 2, hormones and various mediators are released in response to the presence of nutrients in the gastrointestinal tract, which impact the adaptation process [32,38,39]. Growth hormone (GH), a 191-amino-acid peptide, enhances nutrient absorption via mucosal anabolism [22]. Growth hormone exhibits anabolic effects by increasing amino acid transport within the mucosa of the small intestine, enhancing the absorption of nutrients, electrolytes, and water. It positively influences the nitrogen balance in the postoperative period in patient [22,36]. Epidermal growth factor (EGF) is secreted by the salivary glands and Brunner’s glands in the duodenum. It increases DNA synthesis and promotes cell proliferation. EGF actively participates in the healing process of gastric ulcers and helps maintain the proper structure of the intestines. EGF receptors are located in the mucosa of the small intestine. Growth hormone and epidermal growth factor together enhance the height of intestinal villi and increase the transport of glucose, glutamine, and leucine in the jejunum [19,36]. Insulin-like growth factor (IGF-1) is synthesized in the liver and intestinal mucosa in response to oral or enteral nutrition. IGF-1 receptors are present along the entire length of the gastrointestinal tract, stimulating both the small and large intestines, leading to increased absorption of fluids and electrolytes [33]. Glutamine is an important energy source for enterocytes and plays a key role in intestinal adaptation [32]. Intravenous glutamine supplementation prevents villous atrophy caused by total parenteral nutrition. The gastrointestinal tract utilizes approximately 30% of the total glutamine supply, making it a crucial nutrient for the intestines. It promotes intestinal cell proliferation by enhancing the effects of growth factors such as EGF, IGF-1, and TGF-α. Additionally, it activates mitogen-activated protein kinases (MAPKs). Enteral glutamine administration helps maintain crypt cell proliferation in the intestines [32,37,40]. Glucagon-like peptides 1 and 2 (GLP-1 and GLP-2) exhibit higher levels in patients who have undergone extensive small intestine resection while retaining the colon [36]. Both hormones are produced by enteroendocrine L cells located in the distal small intestine and colon. GLP-2 secretion is directly related to food intake, and patients with a terminal ileostomy have impaired postprandial secretion of this peptide. GLP-1 slows gastric emptying and intestinal transit, while GLP-2 increases the absorptive surface of intestinal epithelial cells. Numerous studies in both animals and humans have shown that GLP-2 therapy significantly enhances gastrointestinal adaptation, offering the potential to reduce the need for intravenous nutrient supplementation [36,40,41,42,43].

#### 2.2.5. Advances in Therapeutic Approaches and Surgical Rehabilitation

In recent years, numerous advancements have been reported in intravenous supplementation, surgical techniques, pharmacological treatments, and intestinal transplantation. In the last decade, trophic gastrointestinal hormonal factors have been integrated into intestinal rehabilitation programs for short bowel syndrome (SBS) [44]. The administration of GLP-1 and GLP-2 hormones (or their analogs) is effective in enhancing the natural adaptation process and reducing the need for parenteral nutrition (PN) [9,45,46,47,48,49]. Currently, the only trophic factor approved for clinical use is the glucagon-like peptide-2 (GLP-2) analogue [44]. Surgical rehabilitation procedures for patients with short bowel syndrome (SBS) should focus on minimizing the development of predictable complications, enhancing patient survival, and improving quality of life. The primary goal of surgical rehabilitation for patients with SBS is to increase the absorptive capacity of the remaining intestine. This can be accomplished through procedures aimed at improving intestinal function or expanding the area of absorption. Strategies to enhance absorption include incorporating additional intestine into continuity, relieving mechanical obstruction, or slowing intestinal transit. Intestinal lengthening procedures, designed to expand the absorptive surface and improve function, are viable in selected patients. Intestinal transplantation is used as a last resort when other therapeutic options have not achieved the desired effect. It is one of the methods for regaining full gastrointestinal autonomy [48]. According to the International Intestinal Transplant Registry, between January 1985 and June 2023, a total of 4709 intestinal transplants were performed, of which 2359 were in adults. Despite advancements in surgical techniques and immunosuppressive therapies, intestinal transplantation remains associated with a high risk of complications. Sepsis is the leading cause of death, accounting for 54% of recipient fatalities, followed by rejection at 16% [49]. Is there potential for an additional factor or method to enhance adaptation and promote independence from parenteral nutrition in the management of patients with short bowel syndrome?

## 3. Discussion

### 3.1. Microbiota as Biomarkers of Bowel Adaptation

Intestinal adaptation is a complex process involving both morphological and microbiological changes that compensate for the loss of absorptive tissue. While the structural and functional changes at the epithelial layer following resection, along with their mediators, have been well characterized, the precise mechanisms driving intestinal adaptation remain incompletely understood [20]. Given the complexity of bowel adaptation, traditional markers such as clinical symptoms, histological examination, and metabolic parameters have limitations in accurately monitoring the adaptive process. This has driven interest in identifying non-invasive biomarkers, including the composition and function of the gut microbiota [12]. Due to the rarity of IF, most studies involved small sample sizes and had heterogeneous population characteristics [12]. In healthy individuals, Proteobacteria constitute only a small fraction of the intestinal microbiome (1–2%). However, in patients with intestinal failure, these species often become dominant within the microbial community [12]. Huang et al.’s study demonstrated a dramatic increase in the proportion of Proteobacteria in the SBS II group compared to the control and SBS III group. Additionally, the SBS III group exhibited a significantly higher relative abundance of Bacteroidetes than the control and SBS II group [6]. One study found that the dominance of *Lactobacillus plantarum* spp. was linked to a relatively long duration of parenteral nutrition (PN) before successful weaning, indicating delayed intestinal adaptation [50,51]. In contrast, another study associated this bacterial dominance with a shorter PN duration [12]. Korpela et al. demonstrated that most patients with a high abundance of Proteobacteria were still on PN and had been receiving it for an extended period, while most patients with a high abundance of Clostridium cluster XIVa had stopped PN several years earlier and had only required it for a short time [17]. Huang et al. also showed that a higher abundance of Enterobacteriaceae was linked to a longer duration of PN, whereas a predominance of Lactobacillus was associated with a shorter PN duration [6]. These early findings suggest that patients with intestinal failure (IF) who successfully wean off parenteral nutrition (PN) tend to exhibit a gut microbiome composition more closely resembling that of healthy individuals. This observation implies a potential link between microbial community structure and intestinal functional recovery. In contrast, patients who remain dependent on PN often display persistent dysbiosis, characterized by an overrepresentation of potentially pathogenic taxa such as Proteobacteria and a reduction in beneficial commensals. While these associations do not establish causality, they support the hypothesis that a more balanced or ‘normalized’ microbiota may contribute to, or reflect, improved mucosal function and adaptive capacity. However, further longitudinal and mechanistic studies are needed to clarify whether microbial composition actively influences weaning outcomes or simply mirrors underlying intestinal health [12].

Ossola et al. demonstrated that Lactobacilli were directly associated with stomal output, while Erysipelatoclostridium showed an inverse association. Patients with a stomal output of ≥1500 mL/day had a higher frequency of Lactobacillus and Prevotella, whereas the frequency of Erysipelatoclostridium and Intestinibacter was reduced [8]. Analyzing microbiota-derived metabolite production could offer crucial insights into the functional role of resident gut microbiota. So far, metabolomic studies have been conducted in animal models, a small cohort of pediatric patients, and two adult studies that did not specifically focus on type 1 SBS-CIF patients, with the number of such cases being ≤10 [13,52]. In the small intestine, bacterial fermentation of carbohydrates and lipid catabolism generate gases, organic acids, alcohols, and aldehydes—processes that remain largely unexplored [13,14,16,35,52,53,54,55]. In IF patients receiving PN treatment, changes in the gut microbiome during gut adaptation could potentially serve as biomarkers to determine the optimal timing for transitioning from PN to oral nutrition [12]. To evaluate longitudinal microbiome changes and their metabolic products in IF patients undergoing gradual gut adaptation, prospective studies are needed [12]. According to Cadena M, et al., biomarker serum levels should be assessed within weeks after intestinal resection and monitored over the following months. They recommend measuring these biomarkers at the start of the intestinal adaptation process, when oral feeding begins, and again at six months postoperatively to ensure a meaningful comparison and predictive value [25].

### 3.2. Microbial Imbalance, Fecal Lactate, and Probiotic Interventions

Beyond its role as a biomarker for intestinal adaptation, the gut microbiome may also aid in screening for the risk of D-lactic acidosis. D-acidosis is observed only in certain patients who have undergone extensive small bowel resection with part of the colon in continuity, and some cases have also been reported in patients with a bypass [9,56]. Mayeur et al. propose that the D/L fecal lactate ratio serves as a proxy indicator of microbiome alterations in SBS patients and could help identify those at risk for D-lactic acidosis [34]. In a more recent study, they found that patients with fecal lactate accumulation had an increased presence of lactate-producing bacteria and a reduced proportion of lactate-consuming bacteria, suggesting that microbiome analysis could be useful for detecting individuals prone to lactate buildup [9,12,34]. These findings are supported by the recent review by Chowdhury et al., which emphasizes the critical role of the intestinal microbiome in SBS. The authors note that such microbial imbalances are a hallmark of SBS-related dysbiosis and may significantly impair metabolic homeostasis and adaptation. They also propose the D/L fecal lactate ratio as a promising non-invasive biomarker to identify patients at risk of metabolic complications. Furthermore, their review highlights growing evidence for the use of probiotic and synbiotic interventions aimed at restoring microbial balance, suppressing pathogenic overgrowth, and supporting mucosal adaptation and nutrient absorption in SBS patients [57]. Table 3 summarizes the roles of specific bacterial strains in short bowel syndrome (SBS) and their impact on intestinal adaptation.

### 3.3. The Role of Gut Microbiota Preservation and Probiotic Therapy in Intestinal Adaptation Post Small Bowel Resection

To preserve the gut microbiota—which plays an essential role in intestinal function, immune modulation, and adaptation following small bowel resection—the routine use of oral antibiotics for bowel decontamination should be minimized whenever clinically feasible. Antibiotics can significantly disrupt the microbial ecosystem by reducing overall diversity and selectively depleting beneficial commensal species, thereby impairing mucosal integrity and nutrient absorption. In patients with short bowel syndrome (SBS), where microbial balance is already vulnerable due to altered anatomy and physiology, such disturbances may hinder adaptive processes and exacerbate complications such as small intestinal bacterial overgrowth (SIBO) and liver dysfunction. Goulet and Joly [58] emphasize the importance of maintaining microbial diversity in SBS management, noting that an altered microbiota may both reflect and influence clinical outcomes. Thus, antimicrobial strategies in this population should be carefully weighed against the potential long-term consequences on the gut ecosystem [59]. While research on dysbiosis in SBS patients is growing, detailed insights into the role of microbiota in intestinal adaptation and metabolism remain limited and require further investigation. The use of probiotics to support intestinal adaptation in SBS patients is gaining interest, but evidence of their effectiveness is still scarce. Some studies suggest that probiotic and synbiotic treatments can help reduce pathogenic overgrowth and improve growth and nutritional status in SBS patients [60]. However, Chowdhury et al. stress the urgent need for longitudinal studies with standardized 16S rRNA sequencing methods and rigorous control of confounding factors—such as antibiotic exposure and dietary variation—in order to validate microbial biomarkers and develop evidence-based therapeutic strategies tailored to the microbiome profile of SBS patients [57].

Longitudinal studies standardizing 16S rRNA sequencing and controlling for confounders (antibiotics, diet) are urgently needed to validate microbial biomarkers [9].

### 3.4. Limitations of Microbiome Research in Intestinal Failure

Despite the growing interest in the role of gut microbiota in intestinal adaptation, research in this area faces significant methodological challenges that must be addressed to ensure the reliability and reproducibility of findings.

#### 3.4.1. Sampling Methods

A major limitation in microbiome research is the variability of sampling techniques. While fecal samples are commonly used due to their convenience, they often fail to accurately represent the microbial composition of the small intestine, particularly in patients with end enterostomies. Ostomy effluent samples, while more representative of the small bowel environment, are susceptible to contamination from skin flora and environmental microbes. The lack of standardized protocols for sample collection, storage, and processing further complicates comparisons between studies [6,61].

#### 3.4.2. Sequencing Approaches

A critical challenge in microbiome research lies in the selection of sequencing methodologies. While 16S rRNA gene sequencing remains the most widely used approach due to its cost-effectiveness and ability to profile bacterial taxonomy, it provides limited insights into microbial functional potential. In contrast, shotgun metagenomic sequencing offers a more comprehensive analysis, enabling the identification of microbial genes and metabolic pathways. However, its application is constrained by higher costs, greater technical complexity, and the need for advanced bioinformatics tools. The lack of functional data from 16S rRNA sequencing hinders our understanding of how specific microbial communities contribute to intestinal adaptation, highlighting the need for methodological advancements to bridge this gap [12,13].

#### 3.4.3. Confounding Factors

Interpretation of microbiome data in short bowel syndrome is complicated by numerous confounding variables that can significantly influence gut microbial composition. Factors such as antibiotic administration, the composition of parenteral nutrition, enteral feeding protocols, and dietary intake all have substantial impacts on the microbiota. Many studies fail to adequately control for these variables, which complicates the attribution of observed microbial changes specifically to intestinal adaptation processes. Furthermore, the use of probiotics, prebiotics, or other microbiota-modulating interventions is often inconsistently reported or lacks standardization across study cohorts, further limiting the comparability and interpretability of results. Addressing these confounders through rigorous study design and standardized reporting is essential to advance our understanding of microbiota dynamics in SBS and their clinical implications [35,44].

#### 3.4.4. Cohort Heterogeneity and Study Design

Most available studies are limited by small sample sizes and heterogeneous patient populations, including differences in underlying disease, length and location of resected bowel, and presence or absence of the colon. This heterogeneity reduces the generalizability of findings and increases the risk of type I and type II errors. Longitudinal studies are rare, and most data are derived from cross-sectional analyses, which cannot establish causality [57,62,63].

#### 3.4.5. Recommendations for Future Research: Key Challenges and Future Directions

##### Need for Standardized Protocols in Sample Collection and Processing

Sampling Site and Method:

Microbial communities differ significantly along the gastrointestinal tract. While fecal samples are easy to obtain, they may not accurately represent the small intestine microbiota, which is especially relevant in SBS. More informative sampling methods, such as ostomy effluent collection or mucosal biopsies, require standardized, contamination-minimizing procedures [6,61].

Storage and Handling:

Factors such as delayed freezing, temperature fluctuations, and inconsistencies in DNA extraction can significantly alter microbial profiles. Developing and implementing standardized operating procedures is essential to ensure data reliability.

Future Outlook:

Consensus guidelines—similar to those by the Human Microbiome Project or the International Human Microbiome Standards—should be adapted specifically for SBS and intestinal failure populations to improve reproducibility and comparability across studies.

##### Leveraging Multi-Omics Approaches

While 16S rRNA gene sequencing offers taxonomic resolution, it provides limited insight into microbial function. A multi-omics strategy is essential to unravel the functional impact of microbiota on intestinal adaptation [12,13].

Future Outlook:

Integrating multi-omics data using advanced bioinformatics and machine learning tools will enable the identification of microbial functions and metabolites that drive adaptation and represent potential therapeutic targets.

##### Rigorous Control of Confounding Variables

SBS patients present a range of clinical complexities—frequent antibiotic use, varying nutritional interventions, and inconsistent probiotic administration—that can obscure microbiome-related findings [35,44].

Future Outlook:

Studies must carefully document and control for antibiotics, nutrition, probiotic use, and other potential confounding factors through thoughtful study design (e.g., matched controls, stratification) and robust statistical analysis to accurately isolate microbiome changes linked to intestinal adaptation.

##### Need for Larger, Multicenter, Longitudinal Studies

Most current SBS microbiome studies are limited by small sample sizes, population heterogeneity, and cross-sectional designs, restricting the ability to draw causal inferences.

Longitudinal Designs:

Monitoring patients over time during different stages of adaptation can reveal dynamic microbial changes and their clinical correlates.

Multicenter Collaboration:

Enables recruitment of larger, more diverse cohorts to improve generalizability and statistical power.

Standardized Data Collection:

Harmonizing metadata, microbiome sampling protocols, and outcome definitions across centers is essential for integration and comparison.

Future Outlook:

Creating international consortia focused on SBS and microbiome research will facilitate robust, collaborative efforts to validate microbial biomarkers and therapeutic targets.

##### Validation of Microbial Biomarkers and Causal Mechanisms

To harness the microbiome’s diagnostic and therapeutic potential in SBS, identifying and validating microbial biomarkers is critical.

Biomarker Validation: Requires replication in independent, diverse patient cohorts.

Mechanistic Studies: In vitro models, germ-free animal experiments, and intestinal organoids are essential for elucidating causal pathways between microbes/metabolites and host physiology.

Future Outlook:

Integrating observational studies with mechanistic research will enhance the translational impact and guide the development of personalized microbiome-based interventions.

## 4. Conclusions

Extensive small bowel resection profoundly impacts the structure and function of the gastrointestinal tract, necessitating complex adaptive processes to maintain nutritional balance. Among the various factors influencing intestinal adaptation, the gut microbiota has emerged as a critical yet underexplored component. Alterations in microbiota composition—particularly the rise in Proteobacteria and reduction in beneficial Firmicutes—are increasingly recognized as both consequences of resection and potential modulators of adaptation outcomes. Evidence suggests that patients with microbial profiles closer to those of healthy individuals demonstrate a greater likelihood of successful weaning from parenteral nutrition, whereas dysbiosis is often linked to prolonged PN dependence, metabolic complications, and impaired growth, especially in pediatric populations. Despite these insights, research on microbiome shifts post-resection remains limited in scope, with small sample sizes and variable methodologies. The use of gut microbiota as a biomarker for monitoring the adaptation process holds significant promise, offering a non-invasive and dynamic tool to guide clinical decisions. Furthermore, therapeutic strategies targeting the microbiota such as probiotics, prebiotics, and synbiotics may support intestinal recovery, although more robust clinical trials are necessary to establish efficacy, safety, and optimal formulations. To move forward, interdisciplinary studies combining microbiology, metabolomics, nutrition, and clinical outcomes are essential. Longitudinal analyses involving diverse SBS patient populations will help unravel the intricate interplay between the host and its microbial inhabitants. Understanding and modulating this relationship could pave the way for personalized, microbiota-guided therapies aimed at enhancing intestinal adaptation and improving long-term quality of life for patients with short bowel syndrome.

## Figures and Tables

**Table 1:** Factors and mediators influencing intestinal adaptation [32,38,39].

Factor	Key Mediators/Hormones/Processes
Extent and Location of Resection (presence of colon and ileum)	Presence of colon and ileum significantly influences adaptation capacity. The colon can salvage calories via fermentation and fluid absorption.
Patient’s Age	Younger age is associated with better adaptive capacity.
Pharmacological Treatment	Use of growth factors and medications that stimulate adaptation (e.g., GLP-2 analogues, antisecretory drugs).
Nutritional Therapy	Enteral nutrition stimulates mucosal hyperplasia, gastrointestinal hormone secretion, and pancreaticobiliary secretions.
Hyperphagia	Increased oral intake promotes adaptation by providing luminal nutrients.

**Table 2:** Key mediators [32,38,39].

Mediator	Role in Adaptation
GLP-2 (Glucagon-like Peptide-2)	Stimulates mucosal growth, slows intestinal transit, enhances absorption.
IGF-1 (Insulin-like Growth Factor-1)	Promotes mucosal proliferation and nutrient absorption.
EGF (Epidermal Growth Factor)	Stimulates epithelial cell proliferation and repair.
TGF-α (Transforming Growth Factor-alpha)	Supports mucosal healing and growth.
PYY (Peptide YY)	Slows gastric emptying and intestinal transit, enhancing absorption.
GH (Growth Hormone)	Enhances intestinal growth and function.
GLP-1 (Glucagon-like Peptide-1)	Modulates motility and insulin secretion, contributes to adaptation.
Secretion of Pancreatic Juice and Bile	Facilitates digestion and nutrient absorption, trophic effects on mucosa.

**Table 3:** Summary of the roles of specific bacterial strains in SBS and their impact on intestinal adaptation [6,9,10,12,14,17,34,54].

Bacterial Strain	Impact on SBS/Intestinal Adaptation
Proteobacteria	Dominant in intestinal failure patients; associated with longer duration of parenteral nutrition (PN); often pathogenic.
Enterobacteriaceae	Increased abundance linked to longer PN duration.
Lactobacillus (e.g., *L. plantarum*)	Dominance associated with both shorter and longer PN duration (conflicting findings). High abundance linked to increased stomal output. May help reduce pathogenic overgrowth and support growth.
Clostridium cluster XIVa	Presence associated with earlier PN weaning and shorter PN duration.
Bacteroidetes	Higher relative abundance in SBS group III patients; potentially beneficial.
Prevotella	Increased in patients with high stomal output (≥1500 mL/day).
Erysipelatoclostridium	Inversely correlated with stomal output (lower abundance with high output).
Intestinibacter	Reduced abundance in patients with high stomal output.
Lactate-producing bacteria	Increased presence in patients with fecal lactate accumulation, associated with risk of D-lactic acidosis.
Lactate-consuming bacteria	Decreased presence in patients with fecal lactate accumulation, contributing to D-lactic acidosis risk.

## Abbreviations

The following abbreviations are used in this manuscript:

CIF	Chronic intestinal failure
EGF	Epidermal growth factor
GH	Growth hormone
GLP-1	Glucagon-like-peptide-1
GLP-2	Glucagon-like-peptide-2
IF	Intestinal failure
IGF-1	Insulin-like growth factor
MAPKs	Mitogen-activated protein kinase
PN	Parenteral nutrition
PYY	Peptide YY
SBS	Short bowel syndrome
TGF-α	Transforming growth factor-alpha
